# A novel *VPS13B* mutation in Cohen syndrome: a case report and review of literature

**DOI:** 10.1186/s12881-020-01075-1

**Published:** 2020-06-30

**Authors:** Sara Momtazmanesh, Elham Rayzan, Sepideh Shahkarami, Meino Rohlfs, Christoph Klein, Nima Rezaei

**Affiliations:** 1 Research Center for Immunodeficiencies, Pediatrics Center of Excellence, Children’s Medical Center, Tehran University of Medical Sciences, Tehran, Iran; 2 Network of Immunity in Infection, Malignancy and Autoimmunity (NIIMA), Universal Scientific Education and Research Network (USERN), Tehran, Iran; 3 International Hematology/Oncology of Pediatric Experts (IHOPE), Universal Scientific Education and Research Network (USERN), Tehran, Iran; 4 Department of Pediatrics, Dr. von Hauner Children’s Hospital, University Hospital, LMU Munich, Lindwurmstrasse 4, 80337 Munich, Germany; 5 Department of Immunology, School of Medicine, Tehran University of Medical Sciences, Tehran, Iran

**Keywords:** Cohen syndrome, Neutropenia, Frameshift mutation, Vesicular transport proteins, *VPS13B* protein

## Abstract

**Background:**

Cohen syndrome, an autosomal recessive syndrome, is a rare syndrome with diverse clinical manifestations including failure to thrive, hypotonia, hypermobile joints, microcephaly, intellectual disabilities, craniofacial and limb anomalies, neutropenia and a friendly character. It is associated with mutations of the vacuolar protein sorting 13 homolog B (*VPS13B*) gene, which is involved in the development of the ocular, hematological and central nervous systems. This gene encodes a transmembrane protein playing a crucial role in preserving the integrity of the Golgi complex. To date, more than 150 mutations of *VPS13B* have been reported in over 200 Cohen syndrome patients. Missense or nonsense mutations are the most common mutations.

**Case presentation:**

A 4-year-old girl, born to consanguineous parents, was referred to the pediatric clinical immunology outpatient clinic for investigation of recurrent neutropenia with a history of recurrent infections in the past year. On physical examination, she had the characteristic facial features of Cohen syndrome, developmental delay and speech disorder. She had a cheerful disposition, and her mother gave a history of feeding difficulties in her first months of life. She did not present any ophthalmologic or cardiac abnormalities. Her lab results revealed moderate neutropenia. Serum IgG, IgM, IgA and IgE levels were normal. She fulfilled the clinical diagnostic criteria for Cohen syndrome. WES revealed a novel homozygous frameshift variant in *VPS13B* (LRG_351t1: c.7095del; p.Ser2366AlafsTer49). Currently, she is not experiencing any severe problem, and she undergoes irregular medical treatment once her neutrophil count decreases under the normal limit. Her verbal and motor abilities have improved as a result of speech and occupational therapies.

**Conclusion:**

We reported a novel homozygous frameshift variant in *VPS13B* (LRG_351t1: c.7095del; p.Ser2366AlafsTer49) in a 4-year-old girl with Cohen syndrome. Cohen syndrome should be considered in differential diagnosis of any child with intellectual disability and neutropenia.

## Background

Cohen syndrome (CS) (OMIM No. # 216550), a rare autosomal recessive syndrome, was first reported in 1973 by Cohen and his colleagues [1, 2]. Cohen syndrome has been reported in more than 200 cases to date. Patients with this syndrome manifest characteristic facial features together with psychomotor developmental delay. This syndrome involves ocular, hematologic, musculoskeletal, nervous, gastrointestinal, cardiovascular, and endocrine systems [3–7].

Missense or nonsense mutations in the Vacuolar protein sorting 13 homolog B (*VPS13B*) gene are the cause for Cohen syndrome with more than 150 known variants reported in The Human Gene Mutation Database [8–10]. Members of the VPS13 protein family are all involved in membrane fusion events and vesicular transport mechanisms. While mutations in VPS13B cause CS, the family members VPS13A and VPS13C cause an autosomal recessive Huntington’s-like neurodegenerative disease chorea acanthocytosis [11] and a Parkinson’s-like syndrome, respectively [12].

VPS13B, an essential protein for maintaining the integrity and function of the Golgi apparatus [7], is a large protein with > 4000 amino acids having lipid binding capacity [13, 14]. It is likely that different protein-protein interactions intrinsic to the *VPS13* domain architectures are the reason for the diverse human disease manifestations caused by the *vps13* gene family.

We reported a novel homozygous frameshift variant in the *VPS13B* gene in a girl born to a consanguineous family with recurrent infections and neutropenia, which are rarely reported as initial manifestations of CS. In addition to our case report, we conducted a mini reviewed of the current literature on CS.

## Case presentation

**Fig. 1:**
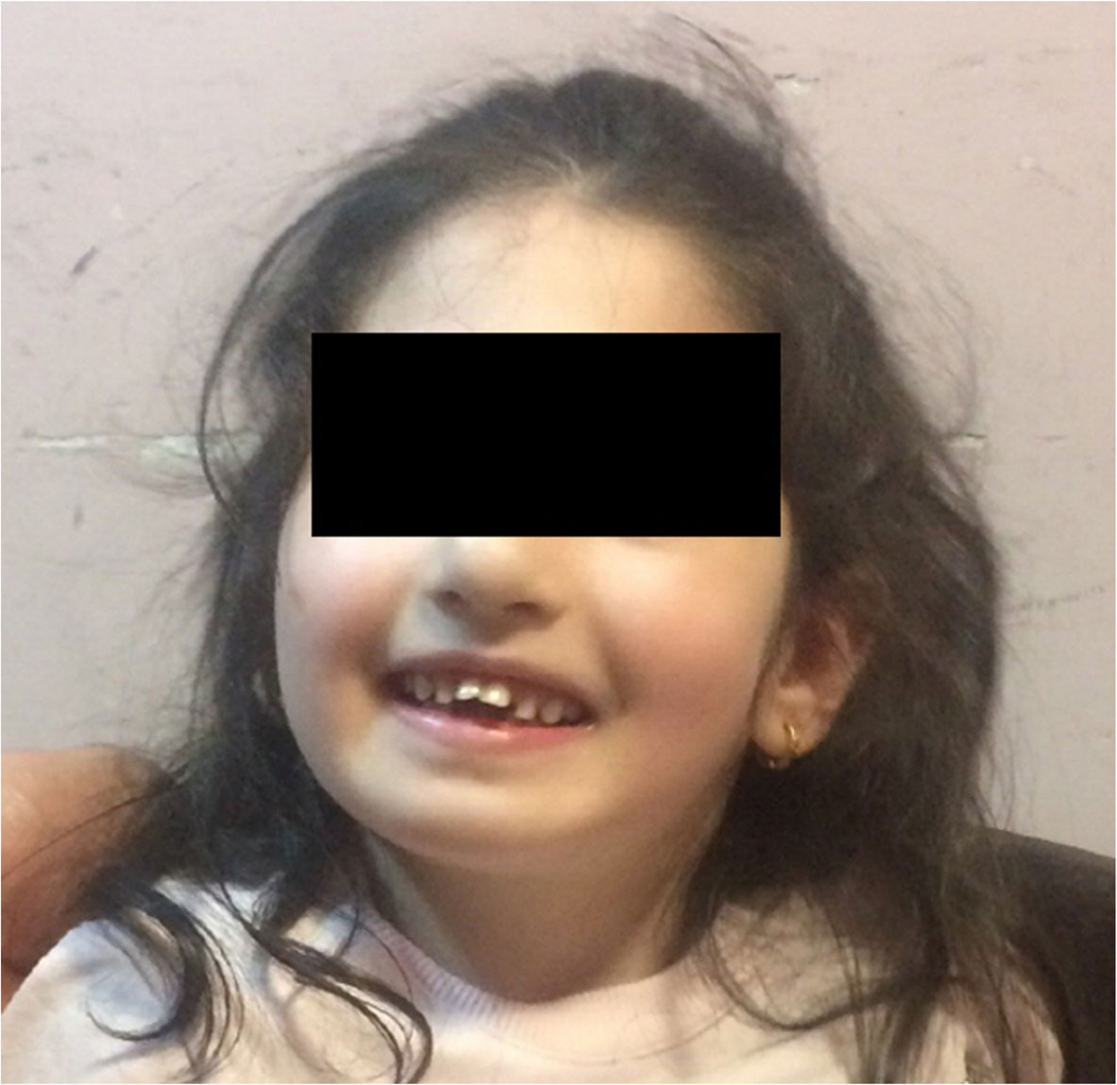
Low hairline, short philtrum, prominent upper central incisors, wave-shaped eyelids, thick and long eyelashes and prominent root of nose in the patient with Cohen syndrome

A four-year-old girl born to a consanguineous family was referred to the pediatric clinical immunology outpatient clinic for diagnostic workup of recurrent infections and recurrent neutropenia in the past year. The medical history, indicated a delay in motoric milestones and feeding difficulties in her first months of life. Currently, she is not able to eat solid food. She had no history of seizures. Her parents were disease free and none of her relative had experienced similar symptoms, she has no siblings. Her mother had a history of stillbirth in her first pregnancy. On clinical examination, the patient had microcephaly, hypotonia, hypermobile joints, motor developmental delay, slender fingers, and a cheerful disposition. She had speech delay and had just started to utter some words. Her facial characteristics included low hairline, short philtrum, prominent upper central incisors, wave-shaped eyelids, thick and long eyelashes and prominent root of nose (Fig. 1). Unlike many other cases of CS [15], the eye examination was normal.

In review of her lab results in the period of past 6 months, absolute neutrophil count ranged from 0.5–2.32 (10^3^/*μL*), neutrophil percentile ranged from 7.8–30%, and other components of complete blood count were normal. Erythrocyte sedimentation rate (ESR), serum IgG, IgM, IgA and IgE levels (assessed with ELISA test), serum T3 and T4, TSH, urea, creatinine and fasting blood sugar were normal. Serum alkaline phosphatase was lower than normal (135 U/L). Table 1 indicates the summary of the patient’s lab results.

**Table 1:** Summary of the patient’s lab results

	At age of 4 years	One month after the first visit	two months after the first visit	six months after the first visit
W.B.C.	5.9 10^3^/μL	5.0 10^3^/μL	7.58 10^3^/μL	4.73 10^3^/μL
Neutrophils count	**0.5 10**^**3**^/**μL**	**0.6 10**^**3**^/**μL**	**2.32 10**^**3**^/**μL**	**0.51 10**^**3**^/**μL**
Lymphocytes count	5.1 10^3^/μL	4.1 10^3^/μL	4.10 10^3^/μL	3.83 10^3^/μL
Monocyte count	0.2 10^3^/μL	0.2 10^3^/μL	1.11 10^3^/μL	0.32
Eosinophil count	0.1 10^3^/μL	0.05 10^3^/μL	0.04 10^3^/μL	0.01
Basophil count	0.0 10^3^/μL	0.01 10^3^/μL	0.01 10^3^/μL	0.01 10^3^/μL
Neutrophil percentage	**7.8%**	**11.5%**	**30.7%**	**10.7%**
Lymphocytes percentage	**86%**	**83.6%**	**54.1%**	**81.0%**
IgG	576 mg/dL			
IgM	125 mg/dL			
IgA	37 mg/dL			
IgE	10.3 IU/mL			
T4	8.4 μg/dL			
T3	154.3 μg/dL			
TSH	1.21 μIU/mL			
Non-fasting blood Glucose	86 mg/dL			
Urea	24 mg/dL			
Creatinine	0.37 mg/dL			
**Alkaline Phosphatase**	**135 U/L**			
Calcium	10.3 mg/dL			
Phosphate (inorganic)	4.8 mg/dL			
ESR first hour	17 mm/hr			

*Abbreviations*: *WBC* White blood cells, *Ig* Immunoglobulin, *TSH* Thyroid-stimulating hormone, *ESR*: Erythrocyte sedimentation rate

Given the patient’s history, physical examination, and lab results, different subtypes of congenital neutropenia, including G6PC3 deficiency, cartilage-hair hypoplasia, ELANE-Related neutropenia and WAS-Related disorders were considered as differential diagnosis. Based on her developmental delay, friendly disposition and facial features, CS was our primary diagnosis. However, due to expenses of Whole exome sequencing in the patient’s country, the test was performed in a foreign country and confirming the final diagnosis took longer than anticipated.

Whole exome sequencing for the patient was performed at the Dr. von Hauner Children’s hospital NGS facility using Agilent V6 + UTR library preparation and an Illumina NextSeq 500 sequencing platform. The bioinformatics analysis pipeline uses Burrows-Wheeler Alingner (BWA 0.7.15). Genome Analysis Tool Kit (GATK 3.6), Variant Effect Predictor (VEP 89) and frequency filters with public and in house databases (e.g. ExAC [16], GenomAD [17] and GME [18]). We found a novel homozygous frameshift variant in the *VPS13B* gene: *VPS13B* (LRG_351t1: c.7095del; p.Ser2366AlafsTer49). Based on the classical 5-tiered system introduced by the American College of Medical Genetics and Genomics, this variant is classified as a pathogenic variant and there is very strong evidence for its pathogenicity [19, 20]. This mutation confirmed our early diagnosis.

Currently, the patient is not experiencing any severe problem. No unanticipated adverse effect has been observed. The patient undergoes irregular medical treatment with 300 μg/injection granulocyte colony stimulating factor (GCSF) once her neutrophil count decreases under the normal limit. Also, Co-Trimoxazole 80 mg/400 mg has been prescribed for infection prophylaxis. Her eye examination remained normal. Her verbal and motor abilities have improved as a result of speech and occupational therapies. She is able to say some simple words like mom and dad. She is also able to walk with her parents’ assistance. Lastly, due to the high likelihood of developing ocular abnormalities, the patient is evaluated by an ophthalmologist periodically.

## Discussion and conclusion

We reported a novel homozygous frameshift variant in the *VPS13B* gene (LRG_351t1: c.7095del; p.Ser2366AlafsTer49), leading to loss of function, in a 4-year-old girl with CS born to a consanguineous heterozygous family of Iranian descent.

In this case, the patient’s most prominent manifestations were intellectual disability and neutropenia. Unlike most of other cases, our patient did not present any ophthalmologic abnormality. She also did not present any cardiologic abnormality, which may be seen in some cases of CS. Thus, the combination of intellectual disability and neutropenia can be a red flag for CS.

Cohen and his colleagues reported CS in two siblings and an isolated patient for the first time in 1973 [1, 2]. Even though they did not indicate neutropenia as a key component of this syndrome, later in 1984, Norio et al. revealed that neutropenia is one of the major findings in these patients [21]**.** The manifestations of this syndrome may be widely varied; however, the Finnish cases are reported to present similar phenotypes [22]. The heterogeneous presentation makes the diagnosis of this syndrome tricky. Nowadays, it is believed that “Mishosseini-Holmes-Walton syndrome” is, in fact, CS [5].

The mutant gene in CS, *VPS13B* (also known as *COH1*), is located on chromosome 8q22.2 [5]. VPS13 plays an important role for several cellular functions, e.g. (1) preserving the integrity and function of the Golgi apparatus, (2) protein glycosylations, and (3) endosomal-lysosomal trafficking [5, 9, 23, 24].

To date 173 *VPS13B* variants have been reported,157 of which 157 are associated with CS [10]. Patients’ different phenotypes are explained by different *VPS13B* variants. Recurrent neutropenia in CS can be caused by a mutation in the *VPS13B* gene, which is linked to increased apoptosis of neutrophils and decreased expression of SerpinB1, which is a vital element for survival of neutrophil [25].

Table 2 summarizes Cohen syndrome’s clinical manifestations. Table 3 illustrates the paraclinical findings in this syndrome.

**Table 2:** Summary of Cohen syndrome’s clinical manifestations [1, 3–5, 22, 25–29]

Common	Less common
**Craniofacial characteristics:**	**Musculoskeletal system:**
• Wave-shaped eyelids • Short philtrum • Thick hair • Low hairline • Long or thick eyelashes • Prominent root of nose • Thick eyebrow • Prominent upper central incisors • High or narrow plate • Microcephaly • Small or absent lobuli of ears	• Short stature • Mild syndactyly • Kyphoscoliosis • Cubitus valgum • Truncal obesity • Simian creases • Lumbar lordosis
	**Ocular system:**
	• Downslanting palpebral fissures
	**Cardiovascular system:**
**Growth and Developmental abnormalities:**	• Cardiac systolic murmur • Decreased left ventricular function in older patients • Floppy mitral valve and mitral regurgitation • Dilation in the descending aorta
• Motor developmental delay • Speech delay • Non-progressive mental retardation • Delayed puberty • Low birth weight	
**Musculoskeletal system:**	
• Hypotonia • Hypermobile joints • Slender limbs • Pes planus • Wide gap between the first toe and the second toe • Genu valgum	**Endocrine system:**
	• Gonadotropin deficiency • Growth hormone deficiency • Insulin resistance • Non-insulin-dependent diabetes mellitus • Cryptorchidism
**Nervous system:**	
• Motor clumsiness • Brisk reflexes • Cheerful disposition	
**Ocular system**:	
(Ophthalmic abnormalities are mostly seen in patients older than 5 years old and are progressive): • Retinochoroidal dystrophy • Myopia (mostly refractive type)	
**Gastrointestinal system:**	
• Neonatal feeding difficulties	
**Other:**	
• High-pitched voice • Reduced fetal activity

**Table 3:** Paraclinical findings [4, 5, 30, 31]

Common	Less common
• Periods of leukopenia (specially neutropenia) (highly common) • Enlarged corpus callosum on MRI	• Low voltage EEG • ECG (ST-segment depression or T-wave inversion

*Abbreviations*: *MRI* Magnetic resonance imaging, *EEG* Electroencephalography, *ECG* Electrocardiography

Management of CS includes regular monitoring and rehabilitation. Recombinant human granulocyte colony stimulating factor (rHG-CSF) can be used in neutropenia management. For monitoring neutropenia, serial absolute neutrophil count (ANC) is recommended. Moreover, since these patients are prone to develop insulin resistance, blood pressure, fasting blood sugar levels, lipid metabolism, and hemoglobin A1C levels should be monitored annually. Moreover, in adolescence, performing oral glucose tolerance tests every 5 years is recommended. Furthermore, speech and physical therapy can help in improving the speech and motor developmental delay, respectively.

Prognosis of CS suggests a normal life expectancy associated with severe ocular diseases and a higher risk of cardiovascular disorders [4, 6].

Kolehmainen et al. reported that the diagnosis of CS can be established when at least six out of the following eight manifestations are present: (1) facial features of Cohen syndrome as described earlier, (2) developmental retardation, (3) microcephaly, (4) cheerful disposition, (5) hypermobile joints, (6) neutropenia, (7) truncal obesity with slender limbs, (8) chorioretinal dystrophy and/or myopia [8, 26]. In addition to this clinical diagnosis criteria, due to the heterogeneous manifestations, we strongly suggest that genetic assessments should be performed whenever CS is suspected. Notably, enlargement of the corpus callosum on the brain magnetic resonance imaging (MRI) in infancy and early childhood can indicate CS [4].

To conclude, we found a novel mutation in *VPS13B* gene in an Iranian 4-year old girl. She presented neutropenia in addition to motor and speech delay, which can be characteristic features of CS caused by *VPS13B* mutation. This case showed that CS should be considered in differential diagnosis of patients with intellectual disability and neutropenia.

## Data Availability

The variant generated during the current study is available in the ClinVar repository, with an accession number of VCV000916582.1 (https://www.ncbi.nlm.nih.gov/clinvar/variation/916582/). The raw sequence datasets generated during the current study are not publicly available because it is possible that individual privacy could be compromised.

## Abbreviations

VPS13B: Vacuolar protein sorting 13 homolog B
MRI: Magnetic resonance imaging
GCSF: Granulocyte colony stimulating factor
rHG-CSF: Recombinant human granulocyte colony stimulating factor: rHG-CSF
ANC: Absolute neutrophil count
